# Tilmicosin inhibits the infections of currently prevalent porcine reproductive and respiratory syndrome viruses via the downregulation of CD163 expression

**DOI:** 10.1080/21505594.2025.2561831

**Published:** 2025-09-15

**Authors:** Ming Qiu, Xinshuai Li, Shuai Li, Shubin Li, Ningjun Xie, Zhe Sun, Hong Lin, Jianzhong Zhu, Kegong Tian, Nanhua Chen

**Affiliations:** a College of Veterinary Medicine, Yangzhou University, Yangzhou, China; b National Research Center for Veterinary Medicine, Luoyang, China; c International Research Laboratory of Prevention and Control of Important Animal Infectious Diseases and Zoonotic Diseases of Jiangsu Higher Education Institutions, Yangzhou, China; d Comparative Medicine Research Institute, Yangzhou University, Yangzhou, China; e Jiangsu Co-Innovation Center for Prevention and Control of Important Animal Infectious Diseases and Zoonoses, Yangzhou University, Yangzhou, China; f Joint International Research Laboratory of Agriculture and Agri-Product Safety, Yangzhou, China

**Keywords:** Porcine reproductive and respiratory syndrome virus, tilmicosin, antiviral activity, CD163, inflammatory factors

## Abstract

Porcine reproductive and respiratory syndrome virus (PRRSV) has a disastrous impact on the global swine industry. Even though PRRS commercial vaccines can provide homologous protection, broadly effective anti-PRRSV strategies are still in urgent need. Tilmicosin is a semi-synthetic macrolide antibiotic that was noticed to have anti-PRRSV capacity in the field. However, the antiviral activities of tilmicosin against currently prevalent PRRSV isolates have not been systematically evaluated. More importantly, the anti-PRRSV mechanisms of tilmicosin are not clarified. In this study, *in vitro* experiments showed that tilmicosin dose-dependently inhibits the replications of prevalent PRRSV (NADC34-like PRRSV-2, NADC30-like PRRSV-2, HP-PRRSV-2, and PRRSV-1) isolates in different target cells (PAMs and Marc-145). *In vivo* studies supported that tilmicosin inhibits NADC34-like PRRSV-2 replication and reduces pathogenic lesions in weaned piglets. Remarkably, the transcriptomic analysis provided the first clue that PRRSV key receptor CD163 was significantly downregulated by tilmicosin, which was further confirmed by qPCR and WB detections. In addition, anti-inflammatory factors (such as IL-10) were downregulated, while pro-inflammatory factors (such as IL-1β) were upregulated after tilmicosin treatment. Flow cytometric analysis indicated that anti-inflammatory may reverse the downregulation effect of tilmicosin on CD163, while pro-inflammatory mediators can enhance the downregulation effect of tilmicosin on CD163, which leads to increased and decreased replication of PRRSV, respectively. These findings demonstrate that tilmicosin can inhibit PRRSV replication via the reduction of CD163 expression, which provides new insights into anti-PRRSV mechanisms of tilmicosin.

## Introduction

Porcine reproductive and respiratory syndrome (PRRS) is one of the most economically significant swine diseases worldwide [1]. PRRS virus (PRRSV) is a positive-sense single-stranded RNA virus clustering within the family of *Arteriviridae* [2]. PRRSV genome is approximately 15 kb in length, containing a 5’ cap structure, a 5’ untranslated region (UTR), at least 10 open reading frames (ORFs), 3’ UTR, and a 3’ poly (A) tail. ORF1a and ORF1b encode at least 16 nonstructural proteins (nsps), while ORF2 to ORF7 encode at least eight structural proteins. ORF2, ORF3, and ORF4 encode minor envelope proteins (GP2a, GP3, and GP4), which form a disulfide-linked heterotrimer. The interaction between the GP2a-GP3-GP4 heterotrimer and the CD163 receptor is an essential and sufficient step for PRRSV infection [3]. CD163 plays a critical role in PRRSV uncoating [4]. Modulation of CD163 expression may regulate PRRSV infection [5–7], while downregulation of CD163 can inhibit PRRSV infection [8,9]. Moreover, the CD163 knockout pig is completely resistant to PRRSV infection [10]. Nanoantibodies developed against CD163 exhibited broad inhibitory effects on distinct lineages of PRRSV isolates [11]. Noticeably, inflammatory mediators may regulate CD163 expression to influence PRRSV infection [12–14].

PRRSV isolates are divided into two species: PRRSV-1 and PRRSV-2 [15]. PRRSV-2 isolates are predominant in Chinese swine herds, while NADC34-like PRRSV-2, NADC30-like PRRSV-2, and highly pathogenic (HP) PRRSV-2 variants are currently prevalent strains [16–18]. Meanwhile, PRRSV-1 isolates are also increasing in China in recent years [19]. Several modified live vaccines (MLVs) have been developed to combat PRRSV in China, including CH-1 R, R98, JXA1-R, HuN4-F112, TJM-F92, GDr180, and the PC strains [20,21]. These MLVs are derived from the PRRSV-2 lineage 5 (classical PRRSV-2) and lineage 8 (HP-PRRSV-2) strains, which may provide satisfactory homologous protection but confer limited heterologous protection [21]. Both PRRSV-2 lineage 1 (NADC34-like and NADC30-like) and PRRSV-1 isolates are circulating in China, but no homologous commercial vaccines are available against these isolates [22]. In addition to the limited cross-protection, MLVs might also reverse to virulent isolates during the application in swine herds [23]. Therefore, broadly effective prevention and control strategies against PRRSV infection are still urgently needed.

Tilmicosin is a semi-synthetic macrolide antibiotic derived from tylosin B and is licensed to be used as a premix feed additive for swine. Tilmicosin is not only an effective antimicrobial for Gram-positive, Gram-negative, and some intracellular bacteria, but also a potential antiviral agent against PRRSV infection. Previous studies showed that tilmicosin has antiviral activities against classical PRRSV-1 and PRRSV-2 isolates [9,15,17]. However, the antiviral activities of tilmicosin against currently prevalent PRRSV isolates were not systematically evaluated. More importantly, the anti-PRRSV mechanisms of tilmicosin remain elusive.

In this study, we determined the antiviral effects of tilmicosin against currently prevalent PRRSV strains in both pulmonary alveolar macrophages (PAMs) and MA104 derived monkey kidney Marc-145 cells. The antiviral activity of tilmicosin against NADC34-like PRRSV-2 isolate was also evaluated in weaned piglets. More importantly, transcriptomic sequencing and flow cytometry were utilized to explore the potential anti-PRRSV mechanisms of tilmicosin. Our *in vitro* and *in vivo* results supported that tilmicosin can inhibit the infection of currently prevalent PRRSV isolates, which is mechanistically associated with the downregulation of CD163 receptor expression via the modulation of inflammatory mediators.

## Materials and methods

### PRRSV strains, cells, and chemicals

PRRSV strains used in this study were all stored in our laboratories, including NADC34-like PRRSV-2 BJ1805-2 isolate and Marc-145-adapted rBJ-VVL strain, NADC30-like PRRSV-2 SD17–38 isolate and Marc-145-adapted rSD-VVL strain, HP-PRRSV-2 XJ17–5 isolate, HP-PRRSV-2 JXA1-R vaccine strain, and PRRSV-1 SD1291 isolate [19,22]. Marc-145 cells were cultured in Dulbecco minimum essential medium (DMEM, SH30243.01, HyClone, USA) supplemented with 10% FBS. PAMs were maintained in Roswell Park Memorial Institute 1640 medium (RPMI-1640, SH30027.01, HyClone, USA) supplemented with 10% fetal bovine serum (FBS, 03U16001DC, EallBio, China). Tilmicosin soluble powder (10%) was from JiBoss (15385, China). Tilmicosin premix (Pharmaceutical License No. 71, Tilmovet® 20% premix) was from Huvepharma (Belgium). Dexamethasone (DEX) powder (D4902) was from Sigma-Aldrich (USA). Recombinant porcine IL-10 (62000-WNAE) was from Sino Biological Inc (China). PMA (P6741) was from Solarbio (China). Recombinant porcine IL-1β (C600390) was from BBI Life Sciences (China).

### MTT assay

Different concentrations of tilmicosin (10% Tilmicosin Soluble Powder, JiBoss, China) were prepared and added to a 96-well plate seeded with PAMs (1 × 10^6^ cells/well). Each concentration was tested in duplicates. At 24 h post-treatment (hpt), the MTT Cell Proliferation and Cytotoxicity Assay Kit (C0009M, Beyotime, China) was used to detect the cell viability according to the manufacturer’s instructions. Similarly, Marc-145 cells were cultured in a 96-well plate until 80% monolayer confluency. The same method was utilized to analyze the influence of tilmicosin on the viability of Marc-145 cells. Based on the MTT results, the values of CC_50_ and IC_50_ were calculated by the GraphPad Prism as previously described [24].

### Real-time RT-PCR assay

The *in vitro* replication of different PRRSV strains in distinct target cells was measured using a universal PRRSV real-time RT-PCR assay with StepOne Plus Real-Time PCR System (Thermo Fisher Scientific, USA) using HiScript III 1^st^ Strand cDNA Synthesis Kit (+gDNA wiper) (R312–01, Vazyme, China) and 2× Taq Master Mix (P111-03, Vazyme Biotech Co., Ltd, China) [25]. In addition, several host genes identified by transcriptomic sequencing were further evaluated by SYBR-Green-based qPCR assays. Relative quantification of target genes was performed using the 2^−ΔΔCt^ method, and GAPDH was set as the endogenous control as previously described [26]. The primers used in this study are shown in Supplementary Table S1, which were modified from previous studies [26–28].

### Western blotting (WB) analysis

WB was modified from our previous study [19]. In detail, cells were firstly lysed in radio-immunoprecipitation assay (RIPA) buffer (P70100, NCM Biotech, China). Secondly, the protein samples were separated on 12% SDS-PAGE gels and transferred to polyvinylidene fluoride (PVDF) membranes (IPVH00010, Merck Millipore, USA). Membranes were blocked with 5% (w/v) nonfat milk in TBS containing 0.05% Tween (TBST) for 1 h at 37°C. Thirdly, rinsed blots were incubated with mouse anti-PRRSV N protein mAb 15A1 (1:500) [27], CD163 rabbit pAb (16646-1-AP, Proteintech, China), or GAPDH mouse mAb (AC002, ABclonal, China) (1:1000 each) at 37°C for 1 h, followed by incubation with HRP-conjugated goat anti-mouse/rabbit IgG (1:10000 in TBST) (D110087-0100, BBI, China) at 37°C for 1 h. Finally, signals were detected by the ECL chemiluminescent detection system (Tanon, China), and images were captured with the WB imaging system (Tanon, China).

### Plaque assay

The plaque morphology was assessed in Marc-145 cells as previously described [29]. The 100% confluent Marc-145 cells were inoculated with 0.1 multiplicity of infection (MOI) of HP-PRRSV-2 XJ17-5 field isolate or JXA1-R vaccine strain at 37°C for 1.5 h, respectively. Then, the supernatants were removed, and the infected cells were washed with PBS three times. The cells were overlaid with DMEM-2% FBS containing 2% methylcellulose (M0512, Sigma-Aldrich, Spain). At 96 h post-infection (hpi), the agarose gels were carefully removed, and the cells were stained with crystal violet solution for 1 h before counting. NADC34-like PRRSV-2 (BJ1805-2), NADC30-like PRRSV-2 (SD17-38), and PRRSV-1 (SD1291) isolates could not be used for plaque morphology analysis because they cannot infect Marc-145 cells.

### Immunofluorescence assay (IFA)

IFA detection of PAMs was performed as previously described [22]. Briefly, a total of 5 × 10^5.0^ primary PAMs per well was seeded in 24-well plates. At 12 h post-seeding (hps), 0.1 MOI of NADC34-like PRRSV-2 (BJ1805–2) was used for infection. Each group was set in a triplex (3 wells). Group-1 PAMs were set as mock infection control, group-2 PAMs were infected by NADC34-like PRRSV-2, and group-3 PAMs were infected by NADC34-like PRRSV-2 and pretreated with 0, 50, 100, or 150 μg/ml tilmicosin when seeding.

At 24 hpi, one well of PAMs from each group was used for IFA detection, respectively. In detail, the infected PAMs were washed once with PBS and fixed in 4% paraformaldehyde (E672002, BBI, China). Cells were permeabilized with PBS containing 0.5% TritonX-100 (A600198, BBI, China) for 10 min and blocked with PBS containing 1% BSA (A8020, Solarbio, China) for 2 h. PRRSV N protein-specific murine mAb 15A1 (1:500) was utilized as the primary antibody, while Dylight 594 goat anti-mouse IgG (1:1000, 35510, Invitrogen, USA) was used as the secondary antibody [19]. Cellular nuclei were counterstained with 4’,6-diamidino-2-phenylindole (DAPI, C1006, Beyotime, China). The results were visualized with an IX53 inverted fluorescence microscope (Olympus, Japan). The other two wells of PAMs at 24 hpi from each group were submitted to RNA extraction and then submitted to transcriptomic sequencing as described below.

### Transcriptomic analysis

Transcriptomic sequencing was performed in Panomix (China). The sample preparation was described in the above IFA detection in PAMs at 24 hpi. Duplicated wells of PAMs from each group were submitted to RNA extraction, which was firstly validated by IFA and real-time RT-PCR and then submitted to transcriptomic sequencing. Three micrograms of RNA per sample were utilized for RNA-Seq sample preparations. At first, sequencing libraries were produced using NEBNext® Ultra™ RNA Library Prep Kit for Illumina® (NEB, USA), and the samples were index-coded. Secondly, the clustering of each index-coded sample was carried out using TruSeq PE Cluster Kit v3-cBot-HS (Illumina, USA) on a cBot Cluster Generation System. And then, each prepared library was sequenced on an Illumina NovaSeq platform. Clean reads were generated by removing low-quality reads, reads containing poly-N, and reads containing adapters from raw data. Differential expressions were analyzed by the edgeR R package. The enrichments of differential expression genes (DEGs) in Gene Ontology (GO) and Kyoto Encyclopedia of Genes and Genomes (KEGG) pathways were evaluated by the cluster Profiler R package as we previously described [26].

### Animal challenge study

The piglet challenge experiment was approved by the Animal Welfare and Ethics Committee of Yangzhou University with reference number 202,201,003 (The animal experiment was started from Jan. 10^th^ to Jan. 25^th^ in 2022). Nine 5-week-old PRRSV-free piglets (from a single litter) were randomly divided into three groups (three piglets in each group). The piglets were obtained from Qidong Longyu Technology Agricultural Development Co. Ltd and challenged on the third day after arrival. The first group of piglets was inoculated with DMEM as a negative control. Piglets in the second and third groups were intranasally and intramuscularly inoculated with 2 ml 10^5.0^ median tissue culture infectious doses (TCID_50_/ml) NADC34-like BJ1805–2 isolate. In addition, tilmicosin (2 mg/kg feed, Tilmovet® 20% premix, Huvepharma, Belgium) was used as a feed additive for the third group of piglets immediately after arrival (pretreatment) according to the manufacturer’s instructions. Rectal temperature and clinical signs were recorded daily. Sera were acquired at 0, 1, 3, 5, 7, 10, 12, and 14 days post-infection (dpi) to analyze the dynamics of viremia by universal PRRSV real-time RT-PCR [25]. The body weights were recorded at 1, 5, 10, and 14 dpi to determine the weight gain. All piglets survived until 14 dpi and were euthanized via intravenous injection with propofol (10 mg/kg, IV) followed by potassium chloride (40 mmol) as recommended by the Chinese Association for Laboratory Animal Sciences, and then tissue samples were collected for histopathological and immunohistochemical (IHC) examinations.

### Flow cytometric analysis

To detect CD163 expression and PRRSV infection in PAMs, PAMs were transferred into 96-well V-bottom plates (1 × 10^6^ cells/well), treated with indicated reagents (100 ng/ml of DEX, porcine IL-10, PMA, or porcine IL-1β, respectively) for 24 h, and then washed twice in 150 µL PBS as previously described [13,14,30,31]. After staining with fixable vital dye eFluor 780 (65-0865-14, Thermo Fisher Scientific, USA) according to the manufacturer’s recommendations, cells were incubated with PE-conjugated anti-pig CD163 antibody (clone 2A10/11, Bio-Rad, USA) or isotype-matched control antibody in FACS buffer (PBS containing 0.5% BSA) for 30 min. Subsequently, cells were fixed with 4% paraformaldehyde and permeabilized with Perm/Wash Buffer (554723, BD, USA) and then incubated with FITC-conjugated anti-PRRSV N protein monoclonal antibodies (PRRSV-specific murine mAb 15A1, conjugated in-house with FITC Conjugation Kit, ab102884, Abcam, UK) in Perm/Wash Buffer. Flow cytometry tests were measured using a FACS LSRFortessa (BD Biosciences, USA), and at least 100,000 cells were collected. All samples were gated based on forward scatter (FSC) and side scatter (SSC) to gate out cellular debris or dead cells. Data analysis was processed by FlowJo software (Tree Star Inc., USA).

### PRRSV entry kinetics

The influence of CD163 downregulation by tilmicosin on PRRSV entry in target cells was evaluated as previously described [32,33]. PAMs (5 × 10^5.0^) and Marc-145 cells (1 × 10^5.0^) were seeded in 24-well plates, respectively. After cell adhesion (12 hps), 150 μg/ml tilmicosin was used to pretreat target cells. Target cells were precooled at 4°C for 0.5 h before infection. Pre-cooling HP-PRRSV-2 XJ17-5 isolate (3 MOI) was used to infect PAMs and Marc-145 cells. The infected cells were set at 4°C for 2 h to allow virus attachment without triggering virus internalization. Then, unattached viruses were discarded by cold PBS washing three times. An aliquot of the cells was centrifuged and harvested in TRIpure reagent (RN0101, Aidlab, China) for attachment evaluation (0 h). Meanwhile, fresh RPMI-1640 with 2% FBS and DMEM with 2% FBS, both containing 150 μg/ml tilmicosin, were used to re-suspend the other harvested PAMs and Marc-145 cells, respectively. At 2, 4, 6, 8, and 10 h, cells were treated with 500 μl 0.25% trypsin (S310JV, BasalMedia, China) to discard un-internalized viruses. The cells were centrifuged and harvested for internalization and replication examinations by universal PRRSV real-time RT-PCR and confocal microscope imaging using a laser-scanning confocal microscope (LSCM, Leica SP8, Germany) as we previously described [25,28].

### Establishment of Marc-145 cell line stably expressing porcine CD163

The Marc-145-pCD163 was established as described previously [27]. In detail, the porcine CD163 gene was cloned into the pENTR4 vector containing an eGFP tag. The pCD163-eGFP gene was subsequently transferred into the lentiviral destination vector (pLenti-DEST-CMV) using an LR Clonase^TM^ II Plus Enzyme Mix (12538120, Invitrogen). 293T cells were pre-seeded in a 6-well plate. The lentiviral vector (pLenti-CMV-eGFP-GOI), along with the packaging plasmids pMD2.G (VSV-G envelope) and psPAX2 (gag/pol/rev), was transfected into the 293T cells at a mass ratio of 4:3:1. Viral supernatant was collected at 48 h post-transfection (48 hpt) and stored at −80°C. Marc-145 cells were seeded in a 6-well plate. When cells reached approximately 60% confluency, lentiviral supernatant was added for infection, supplemented with polybrene (H8761, Solarbio, China). The medium was replaced 24 h post-infection (24 hpi). Three days post-infection, puromycin selection was initiated to eliminate non-transduced cells. Surviving polyclonal cell populations were then subjected to subcloning. Individual monoclonal cell lines were picked and cryopreserved. The obtained Marc-145-pCD163 cell line was used to evaluate whether it could rescue PRRSV susceptibility in the presence of tilmicosin.

### Statistical analysis

The methods of Student’s *t*-test, one-way ANOVA, and two-way ANOVA, where applicable, were carried out for statistical evaluations using the GraphPad Prism [29].

## Results

### Tilmicosin inhibits the infection of prevalent PRRSV isolates in target cells

**Figure 1.**
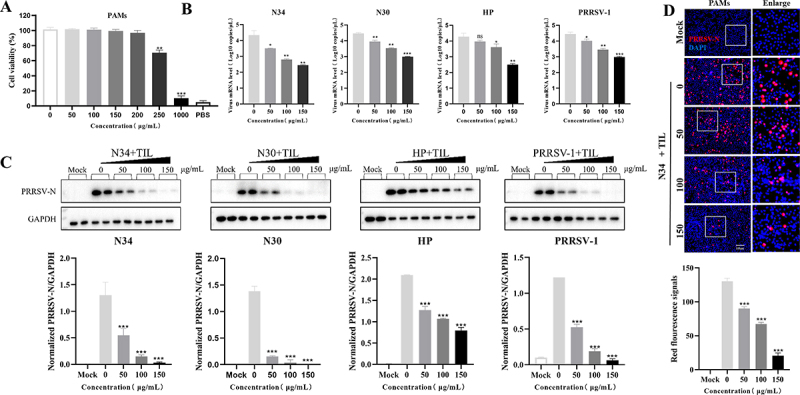
Tilmicosin inhibits currently prevalent PRRSV infection in PAMs. (A) The cytotoxic effect of tilmicosin on PAMs was evaluated at 24 hpi using an MTT assay. (B,C) PAMs were infected by 0.1 MOI of distinct PRRSV isolates (N34, NADC34-like PRRSV-2 BJ1805-2 isolate; N30, NADC30-like PRRSV-2 SD17-38 isolate; HP, HP-PRRSV-2 XJ17-5 isolate; PRRSV-1, SD1291 isolate) and treated with 50–150 μg/ml tilmicosin, respectively. PRRSV ORF7 gene and N protein were subjected to PRRSV universal real-time RT-PCR (B) and WB analyses (C) at 24 hpi [25,27]. (D) NADC34-like PRRSV-2-infected and tilmicosin-treated PAMs were also examined at 24 hpi by IFA using anti-N PRRSV mAb and dylight 594 goat anti-mouse IgG (red). Red fluorescence signals at 24 hpi were analyzed by ImageJ. Statistical significances are denoted by *, *p* < 0.05; **, *p* < 0.01; ***, *p* < 0.001 (*n* = 3). Data shown in the bar graphs are means and SD.

Before we evaluated the anti-PRRSV activity of tilmicosin, the cytotoxicity of tilmicosin was detected in PAMs and Marc-145 at 24 hpt. MTT results in different cells showed that significantly decreased cell viability (*p* < 0.05) was observed when the tilmicosin concentration reached at least 250 μg/ml at 24 hpt (Figures 1(A) and S1A). The CC_50_ values of tilmicosin were 456 μg/mL in PAMs and 526 μg/mL in Marc-145 cells, respectively. To minimize the cytotoxic effects of tilmicosin, 150 μg/ml tilmicosin was used as the highest concentration in our *in vitro* studies.

The anti-PRRSV activity was first tested in Marc-145 cells using HP-PRRSV-2 field isolate (XJ17-5) and vaccine strain (JXA1-R). The plaque assay results showed that tilmicosin could gradually reduce plaque numbers accompanied by increased concentrations (50–150 μg/ml) of tilmicosin, especially at 100 and 150 μg/ml (Figure S1B), which is basically consistent with a previous report that tilmicosin significantly reduced PRRSV replication when the concentration reached 80 μg/ml [34]. Therefore, 50–150 μg/ml tilmicosin was used in the following *in vitro* experiments.

To evaluate the anti-PRRSV activities of tilmicosin on distinct PRRSV isolates in China, representative NADC34-like PRRSV-2 (BJ1805-2), NADC30-like PRRSV-2 (SD17-38), HP-PRRSV-2 (XJ17-5), and PRRSV-1 (SD1291) isolates were used for analyses in PAMs. Real-time RT-PCR results showed that tilmicosin (50–150 μg/ml) dose-dependently reduces viral mRNA copies at 24 hpi in PAMs (*p* < 0.05) (Figure 1(B)). In addition, WB results showed that tilmicosin inhibits N protein expression in PAMs infected by distinct PRRSV isolates in a dose-dependent manner (Figure 1(C)). Moreover, tilmicosin treatment could also dose-dependently reduce NADC34-like PRRSV-2-infected PAMs (indicated by red fluorescence signals) at 24 hpi (Figure 1(D)). The IC_50_ values of tilmicosin against distinct PRRSV isolates ranged from 60 μg/mL to 84 μg/mL (67 μg/mL against NADC34-like isolate, 60 μg/mL against NADC30-like isolate, 84 μg/mL against HP-PRRSV-2 isolate, and 70 μg/mL against PRRSV-1 isolate). Moreover, virus titrations of viral stocks with or without tilmicosin treatment presented no significant difference (Figure S1C), indicating that tilmicosin did not directly kill PRRSV. Overall, these *in vitro* results presented that tilmicosin can inhibit currently prevalent PRRSV isolates in a dose-dependent pattern.

### Tilmicosin relieves NADC34-like PRRSV-2 infection in piglets

**Figure 2.**
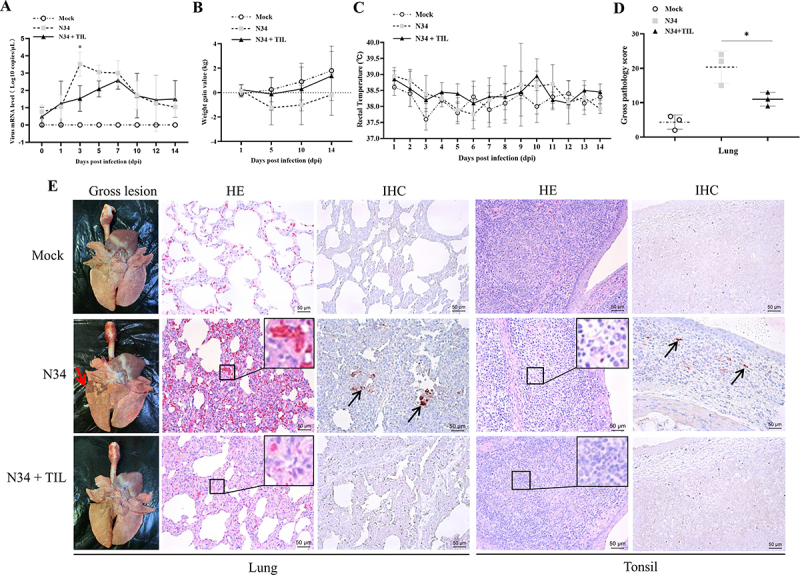
Tilmicosin reduces NADC34-like PRRSV-2 viremia and improves clinical performance in weaned piglets. Piglets in the third (N34 + TIL) group were pretreated using 400 ppm tilmicosin (according to the manufacturer’s instructions) as feed additive since piglet arrival. Piglets in the second (N34) and third groups were intranasally and intramuscularly inoculated with 2 ml 10^5.0^ TCID_50_/ml NADC34-like PRRSV-2 BJ1805-2 isolate at third days after arrival. (A) Viremia was analyzed using PRRSV universal real-time RT-PCR assay [25]. (B) Body weight was determined at 1, 5, 10, and 14 dpi for all piglets in three groups. (C) Rectal temperature was measured daily for 14 dpi. Data shown in the bar graphs are means and SD. Statistical significances are denoted by *, *p* < 0.05 (*n* = 3). (D) The gross pathology of the lungs was scored according to previously described methods [22]. (E) Lung from a representative piglet in mock-infected, NADC34-like PRRSV-2 BJ1805-2 infected (N34), and BJ1805-2 infected and tilmicosin treated (N34 + TIL) groups collected at 14 dpi. Lung consolidation in the N34 group was marked with a red arrow (gross lesion). Representative microscopic pictures of the lung and tonsils were presented, and microscopic changes in N34 and N34 + TIL groups were enlarged (HE). PRRSV-specific antigens were highlighted with black arrows (IHC).

To confirm the antiviral activity of tilmicosin against currently prevalent NADC34-like PRRSV-2 isolates, a pig pretreatment and infection study was performed. Tilmicosin could decrease the viremia from 3 to 7 dpi (significantly different at 3 dpi, *p* < 0.05) compared with the only challenged group (Figure 2(A)). In addition, the weight gain in tilmicosin-treated and NADC34-like PRRSV-2-infected pigs was closer to mock-infected pigs than in only challenged pigs (Figure 2(B)), even though there were no statistical differences among the three groups (*p* > 0.05). NADC34-like PRRSV-2 BJ1805-2 isolate did not cause fever, and there was no significant difference in body temperature among these groups during the animal challenge study (Figure 2(C)).

Overall, lung gross pathology scoring showed that the tilmicosin-treated and NADC34-like PRRSV-2 infected pigs have significantly milder pathological lesions than the only challenged pigs (Figure 2(D)). During the autopsy, obvious lung consolidation could be observed in only NADC34-like PRRSV-2-infected pigs but not in mock-infected or tilmicosin-treated and NADC34-like PRRSV-2-infected pigs (Figure 2(E), lung gross lesion). In addition, histopathological examination identified severe degrees of widened alveolar septum and disseminated congestion in only infected pigs but obviously milder in tilmicosin-treated pigs (Figure 2(E), lung HE). PRRSV antigen was detected in only infected pigs but not in mock-infected or tilmicosin-treated pigs (Figure 2(E), lung IHC). Moreover, histopathological examination of the tonsil could observe the expansion of the germinal center light zone and loss of lymphocytes in the lymphoid follicle in the only infected pigs (Figure 2(E), tonsil HE). The immunohistochemical examination could also identify PRRSV antigen in tonsils from the only challenged pigs (Figure 2(E), tonsil IHC). These *in vivo* results further supported the antiviral activity of tilmicosin against currently prevalent NADC34-like PRRSV-2 isolates.

### Tilmicosin influences the expression of cell surface-related genes in PAMs

To explore how tilmicosin could inhibit PRRSV replication, six libraries from three groups in duplicates were submitted to transcriptomic sequencing. The three groups include mock-infected (NEG), NADC34-like PRRSV-2 infected (POS), and NADC34-like PRRSV-2 and 150 μg/ml tilmicosin treated (TIL) PAMs, respectively. RNA Sequencing (RNA-Seq) generated 6.04 to 10.52 billion clean data (bp) for each sample (Table S2). Quality tests showed that Q20 and Q30 of each sample were higher than 97.26% and 92.72%, respectively. Clean reads mapped to the reference *sus scrofa* genome ranged from 38.14 million (94.66%) to 66.48 million (94.75%), while uniquely mapped reads were from 37.02 million (97.06%) to 64.35 million (96.79%). These results indicated that the RNA-Seq data is qualified and has been submitted to the SRA database with accession No. PRJNA986285.

**Figure 3.**
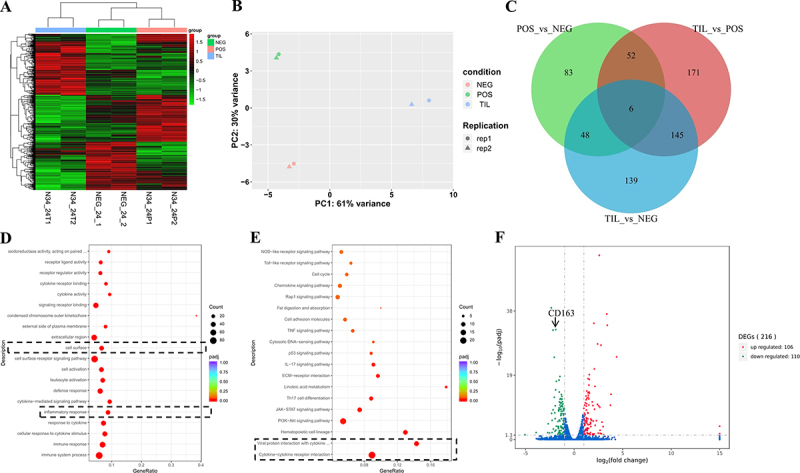
Transcriptomic analysis identifies DEGs associated with cell surfaces. (A) The heatmap of clustered DEGs in mock-infected (NEG), NADC34-like PRRSV-2 BJ1805-2 infected (POS), and BJ1805-2 infected and tilmicosin treated (TIL) PAMs at 24 hpi. Each group was analyzed in duplicates. (B) PCA analysis showed the variances between two replicates of each group of samples. (C) The Venn diagram presented the numbers of specific and shared DEGs in the comparison groups. (D) GO analysis showed the top 20 DEGs in the TIL *vs*. POS comparison. The cell surface and inflammatory response were shown in the dashed line box. (E) KEGG analysis of TIL *vs*. POS comparison identified signal pathways associated with significantly enriched genes. The cytokines-cytokines receptor interaction was shown in the dashed line box. (F) The volcano map showed DEGs in the TIL *vs*. POS comparison at 24 hpi. The significantly downregulated CD163 gene was marked with a black arrow.

RNA-Seq data of NEG, POS, and TIL groups were compared to identify DEGs in POS *vs*. NEG, TIL *vs*. NEG, and TIL *vs*. POS comparisons. The heatmap of clustered DEGs showed that three groups have distinct transcriptomic profiles (Figure 3(A)). The principal components analysis (PCA) showed that the duplication of each sample is satisfied (Figure 3(B)). The Venn analysis showed that 189 and 338 DEGs are detected in POS and TIL groups compared with the NEG group, while there are 374 DEGs between POS and TIL groups. Among these DEGs, 83, 139, and 171 genes were specific for POS *vs*. NEG, TIL *vs*. NEG, and TIL *vs*. POS comparisons, respectively. Six DEGs were shared among three comparison groups (Figure 3(C)). These data suggested that tilmicosin can significantly influence host responses to NADC34-like PRRSV-2 infection in PAMs at 24 hpi.

To evaluate the biological functions of DEGs in TIL vs POS comparison, GO and KEGG enrichment analyses were performed. GO analysis showed that among the TOP 20 significantly enriched gene sets, 19 and 1 of them were clustered within the category of biological process (BP) and cellular component (CC), respectively (Table S3). Most of the significantly enriched DEGs in the categories of BP were associated with immune responses, including inflammatory responses, while the only category of CC was cell surface (Figure 3(D)). KEGG analysis showed that cytokine−cytokine receptor interaction and several RNA sensor-associated signal pathways are significantly enriched (Figure 3(E)). These results suggested that the anti-PRRSV activity of tilmicosin is probably associated with DEGs related to immune responses, inflammatory responses, and the cell surface in PAMs.

### Tilmicosin downregulates CD163 expression in vitro and in vivo

Noticeably, antiviral immune response-related genes (including TLR8, CXCL10, and MX1) were downregulated rather than enhanced after tilmicosin treatment (Figure S2, Table S3), suggesting that the anti-PRRSV activity of tilmicosin is unlikely through the enhancement of antiviral immune responses. In contrast, the decreased antiviral innate immunity may be due to inhibited PRRSV infection by tilmicosin. Intriguingly, the cell surface-associated CD163 gene was also significantly downregulated in the tilmicosin-treated group, indicating that the downregulation of CD163 expression might be correlated with the anti-PRRSV activity of tilmicosin in PAMs (Figure 3(F), Table S3).

**Figure 4.**
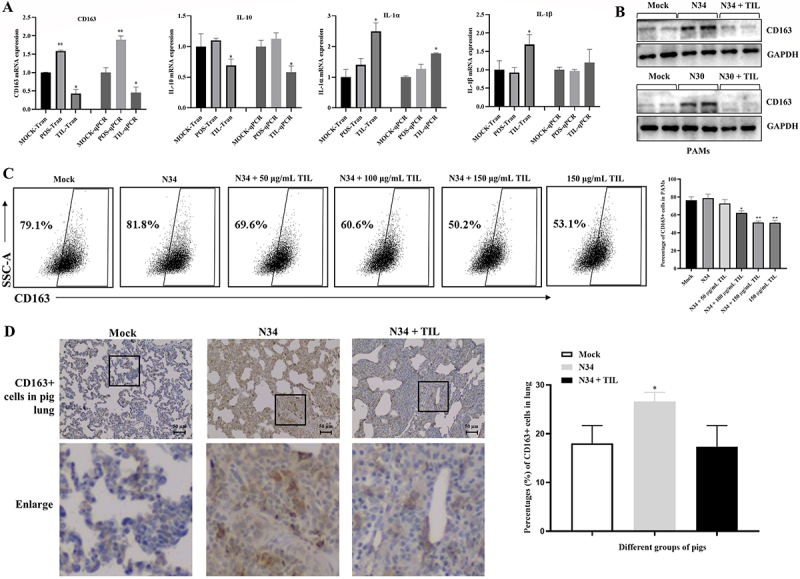
Tilmicosin downregulates CD163 expression *in vitro* and *in vivo*. (A) The mRNA levels of representative CD163, IL-10, IL-1α, and IL-1β genes were compared between the transcriptomic analysis (left) and qPCR detection (right) in PAMs at 24 hpi. (B) WB analysis of CD163 expression in mock-infected, NADC34-like PRRSV-2 BJ1805-2 infected (N34), BJ1805-2 infected and 150 μg/ml tilmicosin treated (N34 + TIL) PAMs or NADC30-like PRRSV-2 SD17-38 infected (N30), SD17-38 infected and 150 μg/ml tilmicosin treated (N30 + TIL) PAMs at 24 hpi (in duplicates). (C) Representative flow cytometric analyses for the effects of tilmicosin (50–150 μg/ml) on CD163 expression in PAMs infected with or without NADC34-like PRRSV-2 BJ1805-2 (N34) at 24 hpi. The percentages of CD163 positive cells in PAMs for all groups were also shown. (D) Representative IHC results of CD163 expression in mock, NADC34-like PRRSV-2 BJ1805-2 (N34) infected, and BJ1805-2 infected and tilmicosin treated (N34 + TIL) pigs. Representative CD163 positive cells were indicated with red arrows. The percentages of CD163 positive cells in lungs from each group of piglets were also analyzed. Statistical significances are denoted by *, *p* < 0.05; **, *p* < 0.01. Data shown in the bar graphs are means and SD.

To verify the RNA-Seq results, representative genes associated with cell surface, inflammatory responses, and immune responses were selected and tested by qPCR. qPCR results were basically consistent with RNA-Seq results (Figures 4(A) and S2). Remarkably, the anti-inflammatory indicator IL-10 was significantly downregulated in tilmicosin-treated PAMs, while pro-inflammatory factors IL-1α and IL-1β were significantly upregulated. WB results confirmed that tilmicosin downregulates CD163 protein expression in PAMs infected by NADC34-like PRRSV-2 and NADC30-like PRRSV-2 (Figure 4(B)). Furthermore, flow cytometric analysis showed that the downregulation of CD163 protein expression by tilmicosin is dose-dependent and independent of PRRSV infection in PAMs (Figure 4(C)).

**Figure 5.**
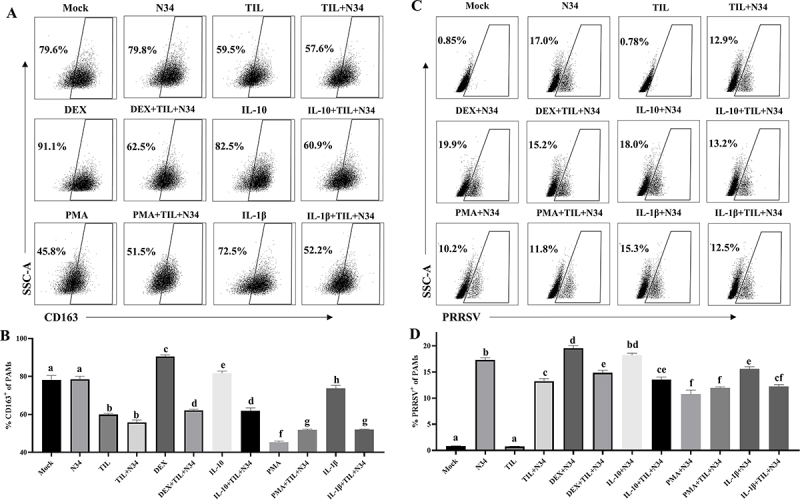
Inflammatory mediators modulate CD163 expression and PRRSV infection. (A) Representative flow cytometric results for the effects of inflammatory mediators on CD163 expression in PAMs at 24 hpi. (B) Percentages of CD163 positive cells in PAMs from all groups. (C) Representative flow cytometric results for the effects of inflammatory mediators on PRRSV infection. (D) Percentages of PRRSV-positive cells in PAMs from all groups. NADC34-like PRRSV-2 BJ1805-2 (N34) isolate (0.1 MOI) was used for PRRSV infection, while 150 μg/ml tilmicosin (TIL), 100 ng/ml of dexamethasone (DEX), IL-10, PMA, and IL-1β were utilized for treatment. The statistical analyses of group comparisons were carried out by the Student’s test (different letters indicate significantly different, *p* < 0.05) (*n* = 3). Data shown in the bar graphs are means and SD.

Moreover, we also evaluated the influence of tilmicosin on CD163 protein expression in NADC34-like PRRSV-2-infected piglets. IHC results showed that CD163 protein expressions are downregulated in lung samples from the TIL group compared with lung samples from the POS group (Figure 4(D)). Noticeably, WB results (Figures 4(B)) showed that CD163 expression obviously increased after PRRSV infection, but it was not detected in flow cytometric analysis (Figures 4(C) and 5(A)), which is likely due to the detection of cell surface CD163 protein in flow cytometric analysis but whole-cell CD163 protein (including intracellular CD163 for uncoating) in WB analysis. Overall, these results validated that tilmicosin downregulates CD163 *in vitro* and *in vivo*.

### Inflammatory factors modulate CD163 expression and PRRSV infection

As shown in Figure 4(A), tilmicosin could downregulate anti-inflammatory factors but upregulate pro-inflammatory factors in PAMs at 24 hpi. To confirm this phenotype, we also detected these inflammatory mediators in Marc-145 cells. Similar to the results in PAMs, tilmicosin also downregulated anti-inflammatory factor (IL-10) while upregulated pro-inflammatory factors (IL-1α and IL-1β) in Marc-145 cells at 24 hpi (Figure S3).

Several inflammatory factors (such as IL-10) might be involved in the regulation of CD163 expression [12,13]. Therefore, we evaluated the influence of inflammatory mediators on CD163 expression and PRRSV infection in PAMs (Figure 5). Anti-inflammatory mediators (Dexamethasone and IL-10) could reverse the downregulation effect of CD163 expression (percentages from 57.6% to 62.5% (*p* < 0.05) and 60.9% (*p* < 0.05)), but pro-inflammatory mediators (PMA and IL-1β) could enhance the downregulation effect of CD163 expression (from 57.6% to 51.5% (*p* < 0.05) and 52.2% (*p* < 0.05)) in tilmicosin treated and PRRSV-infected (TIL + PRRSV) PAMs (Figure 5(A,B)). Furthermore, anti-inflammatory mediators (Dexamethasone and IL-10) might reverse the inhibition of PRRSV infection (percentages from 12.9% to 15.2% (*p* < 0.05) and 13.2% (*p* > 0.05)), and pro-inflammatory mediators (PMA and IL-1β) might further enhance the inhibition of PRRSV infection (from 12.9% to 11.8% (*p* < 0.05) and 12.5% (*p* > 0.05)) in TIL + PRRSV PAMs (Figure 5C,D). These results supported that the downregulation of CD163 expression and inhibition of PRRSV infection by tilmicosin may be achieved by modulating anti- and pro-inflammatory factors.

To confirm the causality between the downregulation of CD163 and the inhibition of PRRSV replication, we established a Marc-145 cell line (Marc-145-pCD163) stably expressing porcine CD163 to evaluate whether pCD163 overexpression could rescue PRRSV susceptibility with the presence of tilmicosin. As shown in Figure S4, the inhibition of PRRSV replication by tilmicosin was nearly blocked in the Marc-145-pCD163 cells. The result supported that the downregulation of CD163 by tilmicosin is a causation for the inhibition of PRRSV replication.

### Tilmicosin may retard PRRSV uncoating to inhibit virus replication

**Figure 6.**
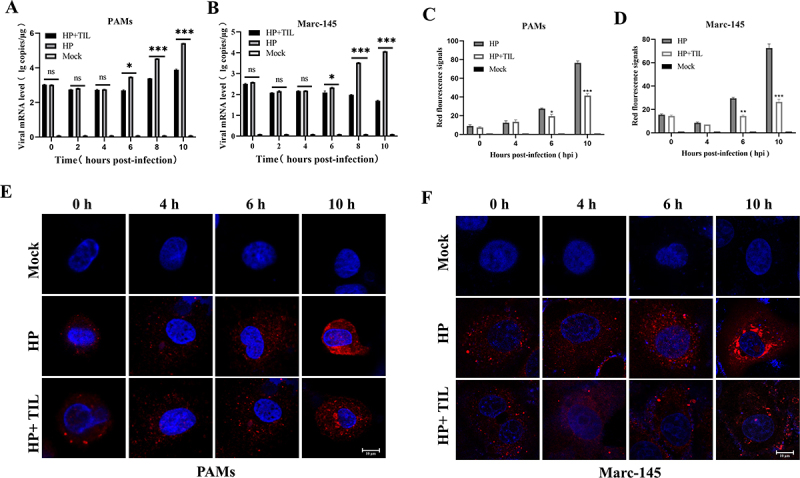
Tilmicosin retards the PRRSV uncoating process. (A,B) The effects of 150 μg/ml tilmicosin 12 h pretreatment on PRRSV (HP-PRRSV2 XJ17-5 isolate (HP)) replication from 0 hpi to 10 hpi in PAMs and Marc-145 cells, respectively. (C−F) HP-PRRSV2-infected (3 MOI XJ17-5 (HP)) and tilmicosin-treated PAMs and Marc-145 cells were detected under a laser-scanning confocal microscope at the excitation wavelengths 340 nm and 561 nm, respectively [28]. Cell nucleus was stained with DAPI (blue), and PRRSV N protein was examined using anti-N PRRSV mAb and dylight 594 goat anti-mouse IgG (red). Statistical significances are denoted by *, *p* < 0.05; **, *p* < 0.01 (*n* = 3). Data shown in the bar graphs are means and SD.

To evaluate the influence of CD163 downregulation by tilmicosin on PRRSV entry, we detected the effects of 150 μg/ml tilmicosin pretreatment (12 h before infection) on PRRSV entry and replication from 0 h to 10 h. In PAMs, tilmicosin pretreatment did not influence the attachment and internalization (0–4 hpi) but significantly inhibited viral replication since 6 and 8 hpi (Figure 6(A)). Similarly, tilmicosin pretreatment did not influence attachment and internalization but inhibited virus replication since 6 and 8 hpi in Marc-145 cells (Figure 6(B)). PRRSV uncoating occurred at around 5 hpi as described previously [33]. Confocal results also supported that tilmicosin may retard the PRRSV uncoating process (4–6 hpi) in both PAMs and Marc-145 cells (Figure 6(E,F)). The results suggested that tilmicosin may downregulate CD163 expression to retard PRRSV uncoating and then inhibit virus replication.

## Discussion

Due to the high genetic diversity of PRRSV and limited cross-protection conferred by commercial vaccines, alternative anti-PRRSV strategies are urgently needed [35]. In this study, we confirmed that tilmicosin not only inhibited currently prevalent PRRSV infection in different target cells but also reduced viremia and improved clinical performance in NADC34-like PRRSV-2-infected piglets. More importantly, our results provided the first clue that the anti-PRRSV activity of tilmicosin is mechanistically related to the downregulation of CD163 expression, which may be achieved by modulating anti- and pro-inflammatory factors.

PRRSV is one of the most rapidly evolving RNA viruses [36]. In China, wild-type PRRSV-2 was found in 1995 [37]. In 2006, HP-PRRSV-2 emerged in Chinese swine herds, causing High Fever Disease [18]. Since 2013, NADC30-like PRRSV-2 has become prevalent in Chinese swine herds [38]. In 2017, NADC34-like PRRSV-2 also emerged and became predominant in China [39]. Moreover, wild-type PRRSV-1 has also been identified in China since 2006 [19]. Due to the co-existence of distinct PRRSV isolates, PRRS prevention and control became complicated in China. Molitor first reported that tilmicosin can inhibit PRRSV replication in a dose-dependent manner in PAMs *in vitro* [40]. In addition, tilmicosin could inhibit classical PRRSV-1 (LV strain) and PRRSV-2 (PA8 strain) replication in Z-mac cells [34]. Tilmicosin could also improve clinical performance for nursery pigs artificially infected by PRRSV 94-2367 isolate [41,42]. Here, we evaluated the antiviral activities of tilmicosin against currently prevalent Chinese PRRSV isolates. Our results showed that tilmicosin could effectively inhibit the infection with lineage 1 (NADC34-like PRRSV-2 and NADC30-like PRRSV-2), lineage 8 (HP-PRRSV-2), and subtype 1 PRRSV-1 isolates. These results indicated that the anti-PRRSV activities of tilmicosin are not overwhelmed by the high genetic diversity of PRRSV isolates. Therefore, the antiviral effect of tilmicosin is more likely achieved by modulating the functions of target cells rather than directly acting on PRRSV.

PAMs are the primary target cells for PRRSV replication *in vivo* [2]. Tilmicosin can accumulate in PAMs to a high concentration [43]. We previously hypothesized that tilmicosin might modulate cell functions, such as immune system processes, to resist PRRSV infection. Our transcriptomic results showed that the majority (19 out of 20) of the most significantly changed genes are classified in BP categories correlating with immune responses, including the toll-like receptor signaling pathway. Activation of the TLR7 signaling pathway could inhibit PRRSV infection in PAMs and piglets [44,45]. In addition, TLR7, TLR8, and TLR9 agonists might be used as adjuvants for inactivated PRRSV vaccine [46]. However, tilmicosin significantly downregulated TLR8, MX1, and CXCL10 expression in PAMs, which suggested that the anti-PRRSV activity of tilmicosin is unable to be achieved by enhancing antiviral innate immunity. On the contrary, the downregulated antiviral innate immunity is probably a result of decreased PRRSV infection by tilmicosin.

Our transcriptomic results also showed that the most significantly changed genes clustered within the CC category are associated with the cell surface, including the CD163 gene. CD163 is the essential receptor for PRRSV infection [3]. Downregulation of CD163 expression by microRNA 181 and lipopolysaccharide could inhibit PRRSV infection [8,9]. In this study, we identified for the first time that tilmicosin can downregulate CD163 expression *in vitro* and *in vivo*.

CD163 is a specific marker of macrophages and monocytes. The level of CD163 expression could be regulated by its upstream host factors [47]. CD163 expression can be induced by anti-inflammatory mediators, including glucocorticoids, IL-6, and IL-10, while being inhibited by pro-inflammatory mediators such as TNF-α, IL-1α, and IL-1β [12]. Cryptotanshinone inhibited the activation of anti-inflammatory factor IL-10 to inhibit CD163 expression and protect PAMs from PRRSV infection [13]. A potent steroidal anti-inflammatory mediator dexamethasone might enhance CD163 expression in porcine IPKM immortalized macrophages [14]. Our results showed that tilmicosin significantly influences inflammatory responses. Tilmicosin could downregulate anti-inflammatory factors while upregulating pro-inflammatory factors.

Further evaluation confirmed that anti-inflammatory mediators could reverse the tilmicosin-induced downregulation of CD163 and then increase PRRSV infection. In contrast, pro-inflammatory mediators could enhance the tilmicosin-induced downregulation of CD163 and then decrease PRRSV infection. These results are consistent with a previous study that both anti-inflammatory mediators, dexamethasone and IL-10, could enhance PRRSV-1 (Lena) infection of monocytes [48]. All these results indicated that the anti-PRRSV activity of tilmicosin is mechanistically associated with the downregulation of CD163, which may be achieved by the adjustment of inflammatory factors. What other host factors involved in the inflammatory regulation and anti-PRRSV activity of tilmicosin deserve further investigation.

PRRSV enters PAMs mainly through the receptor-mediated endocytic pathway (such as CD163). PRRSV internalizes via a clathrin-dependent pathway and then transports into endosomes/lysosomes, where the uncoating occurs. Proteolytic enzymes specially processed for lysosomes in the rough endoplasmic reticulum are only biologically active at acidic pH. Acidic pH-induced conformational changes of viral particles trigger further uncoating of the virus to release viral genomic RNA into the cytoplasm [2,34]. The accumulation of tilmicosin in PAMs resulted in the elevation of endosomal pH due to the basic property of the compound, and then the subsequent uncoating process would be inhibited to block PRRSV replication. Remarkably, PAMs are also the target cells for PCV, but tilmicosin did not affect PCV2 replication even though it reduced PRRSV load in the same pigs [42]. Therefore, the unique anti-PRRSV activity of tilmicosin is mechanistically associated with the downregulation of CD163 expression and the retardation of virus uncoating.

This study still has several limitations. The exact anti-PRRSV mechanisms of tilmicosin require comprehensive evaluations in the following studies. Only three piglets used in each group are insufficient to draw robust conclusions. More pigs are needed to evaluate the anti-PRRSV efficacy of tilmicosin in the future. This study mainly focuses on the influences of inflammatory factors on CD163 expression and PRRSV replication and whether other host factors involved in these processes need further clarification.

Noticeably, the field usage of tilmicosin might influence PRRS vaccination efficacy (Figure S1B). More importantly, even though tilmicosin has broad-spectrum anti-PRRSV activity, under the global requirement to reduce the impact of bacterial antimicrobial resistance (AMR), the applications of tilmicosin in the field must be strictly controlled. Whether the wide usage of tilmicosin could accelerate the generation of tilmicosin-resistant PRRSV isolates requires further investigation. In addition, a previous study determined the minimum inhibitory concentration (MIC) values of antibiotics (ceftiofur, enrofloxacin, florfenicol, tulathromycin, and tilmicosin) against swine pathogens (*A. pleuropneumoniae*, *P. multocida*, and *S. suis*). The method is commonly used to determine antimicrobial susceptibility or resistance. Their results showed that tilmicosin has the lowest drug potency against these bacterial organisms based on MIC_90_ values [49]. Moreover, the application of manure containing veterinary antibiotics (such as sulfamethoxazole, tiamulin, and tilmicosin) could lead to the contamination of agricultural soils, which strongly impacted the total prokaryotic and ammonia-oxidizing microorganism communities [50]. Therefore, the abuse of tilmicosin would result in more serious consequences than its benefits.

## Conclusions

This study confirmed the antiviral activities of tilmicosin against currently prevalent PRRSV isolates. Mechanistically, tilmicosin may modulate anti- and pro-inflammatory factors to downregulate CD163 expression and then inhibit PRRSV replication. This study provides new insight into the unique anti-PRRSV mechanisms of tilmicosin. A rational utilization strategy of tilmicosin would benefit PRRSV prevention and control.

## Supplementary Material

Supporting Table S3 Top 20 DEGs in TIL POS.docx

Figure_S2_final.tiff

Figure_S3_final.tiff

Supporting Table S1 primers.docx

Figure_S4_final.tiff

Supporting Table S2 RNASeq quality.docx

Figure_S1_FINAL.tiff

Supplementary_figure_and_table_legends.docx

## Data Availability

The RNA-Seq data in this study can be accessed in the SRA database with accession No.: PRJNA986285 (https://submit.ncbi.nlm.nih.gov/subs/sra/). The data that support the findings in this work are available in the data repository with DOI: 10.5281/zenodo.17089815.
