# Supplementary material for: Tilmicosin inhibits the infections of currently prevalent porcine reproductive and respiratory syndrome viruses via the downregulation of CD163 expression
Source: Virulence. 2025 Sep 15;16(1):2561831. doi: 10.1080/21505594.2025.2561831 (PMC12452467; doi:10.1080/21505594.2025.2561831)
Supplement: Supporting Table S3 Top 20 DEGs in TIL POS.docx [file KVIR_A_2561831_SM3158.docx]

Table S3. Top 20 differential expression genes in the TIL vs POS comparison

| **No.** | **Name** | **id** | **Chromosome** | **baseMean_TIL** | **baseMean_POS** | **foldChange(POS/TIL)** | **log2FoldChange** | **pval** | **padj** |
| --- | --- | --- | --- | --- | --- | --- | --- | --- | --- |
| 1 | CSF3 | gene19348 | NC_010454.4 | 6551.760349 | 1085.879075 | 0.165738522 | -2.593019136 | 2.30111E-59 | 3.5253E-55 |
| 2 | TLR8 | gene26958 | NC_010461.5 | 437.4824599 | 2241.116568 | 5.122757536 | 2.35692061 | 1.5147E-43 | 1.16026E-39 |
| 3 | CYP3A46 | gene5265 | NC_010445.4 | 540.1257268 | 52.32679948 | 0.096878924 | -3.367673342 | 1.51755E-41 | 7.74962E-38 |
| 4 | PLA2G2D | gene11732 | NC_010448.4 | 5805.107556 | 1415.689121 | 0.243869576 | -2.035818307 | 1.13623E-38 | 4.35176E-35 |
| 5 | CEMIP | gene13730 | NC_010449.5 | 737.8272683 | 69.15645364 | 0.09372987 | -3.415347314 | 5.51652E-38 | 1.69026E-34 |
| 6 | CD163* | gene9571 | NC_010447.5 | 8653.935116 | 32046.74 | 3.703140776 | 1.888749395 | 1.27709E-36 | 3.26084E-33 |
| 7 | CSF3R | gene12017 | NC_010448.4 | 375.2212099 | 1694.64018 | 4.516376301 | 2.175165695 | 2.11655E-36 | 4.63223E-33 |
| 8 | EPCAM | gene6525 | NC_010445.4 | 4325.799767 | 806.2889501 | 0.186390724 | -2.423598032 | 2.96713E-32 | 5.68205E-29 |
| 9 | GPR18 | gene18642 | NC_010453.5 | 491.4250868 | 72.25565245 | 0.147032893 | -2.765789151 | 7.40024E-32 | 1.25969E-28 |
| 10 | LIF | gene22622 | NC_010456.5 | 196.7132751 | 9.499193609 | 0.04828954 | -4.372145463 | 2.4354E-28 | 3.73104E-25 |
| 11 | TLR7 | gene26959 | NC_010461.5 | 254.7414451 | 1057.543963 | 4.151440544 | 2.053612036 | 3.49555E-28 | 4.86835E-25 |
| 12 | LGR4 | gene3202 | NC_010444.4 | 775.1754567 | 184.1387294 | 0.237544581 | -2.073729796 | 1.05378E-24 | 1.34532E-21 |
| 13 | CYP51 | gene16966 | NC_010451.4 | 3285.045108 | 1113.121251 | 0.338845043 | -1.561302428 | 5.56128E-23 | 6.55375E-20 |
| 14 | LPL | gene22100 | NC_010456.5 | 3400.516039 | 9377.576867 | 2.757692291 | 1.463461487 | 2.15316E-22 | 2.35617E-19 |
| 15 | SDS | gene22471 | NC_010456.5 | 9744.610465 | 3623.768677 | 0.371874144 | -1.427113652 | 9.94821E-22 | 1.01604E-18 |
| 16 | HMGCS1 | gene24992 | NC_010458.4 | 2184.982151 | 741.0328622 | 0.339148245 | -1.560012067 | 1.6728E-21 | 1.60171E-18 |
| 17 | RSAD2 | gene6882 | NC_010445.4 | 717.8817599 | 2119.975023 | 2.953097768 | 1.56222912 | 3.05246E-21 | 2.7508E-18 |
| 18 | F3 | gene8456 | NC_010446.5 | 224.8584608 | 804.0304139 | 3.575717858 | 1.838232905 | 6.95014E-21 | 5.91534E-18 |
| 19 | HBEGF | gene4930 | NC_010444.4 | 1912.644469 | 666.1769659 | 0.348301515 | -1.521591348 | 1.00635E-19 | 8.11439E-17 |
| 20 | GPR171 | gene21096 | NC_010455.5 | 1864.615697 | 420.7108306 | 0.225628708 | -2.147977453 | 2.50055E-19 | 1.91542E-16 |

* The PRRSV key receptor CD163 encoding gene is highlighted in red.
