# Supplementary figures and images for: Tilmicosin inhibits the infections of currently prevalent porcine reproductive and respiratory syndrome viruses via the downregulation of CD163 expression
Source: Virulence. 2025 Sep 15;16(1):2561831. doi: 10.1080/21505594.2025.2561831 (PMC12452467; doi:10.1080/21505594.2025.2561831)

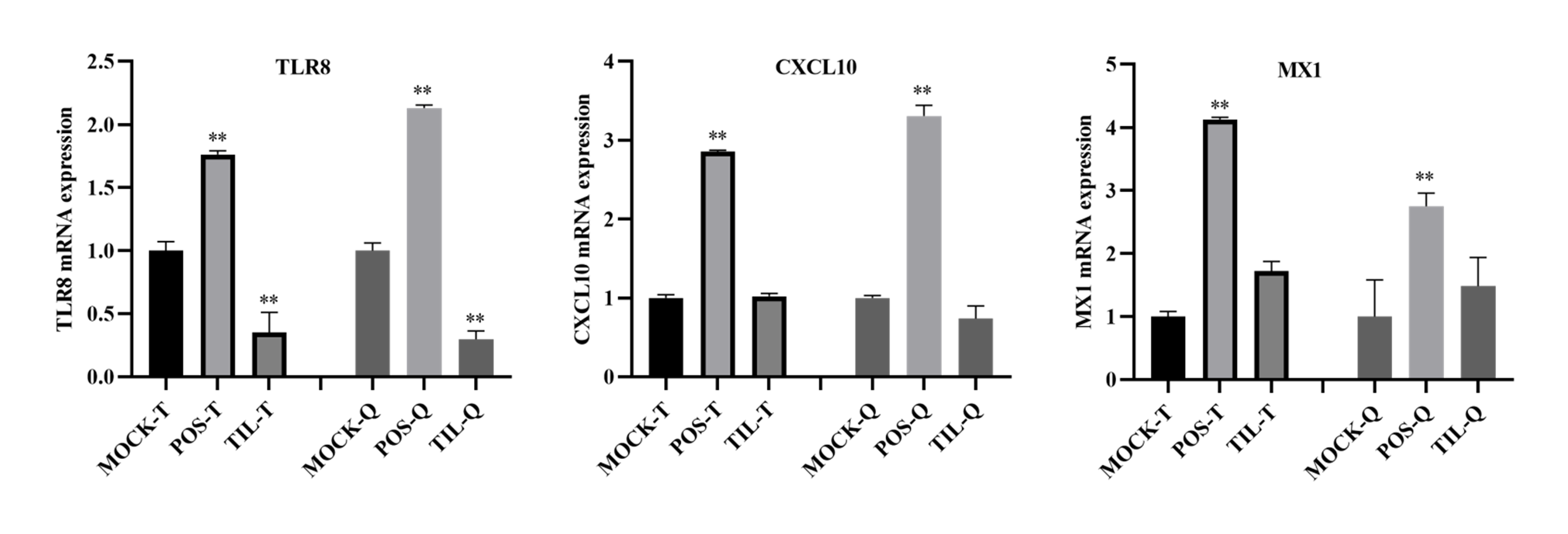

Supplement: Figure_S2_final.tiff [file KVIR_A_2561831_SM3157.tiff]

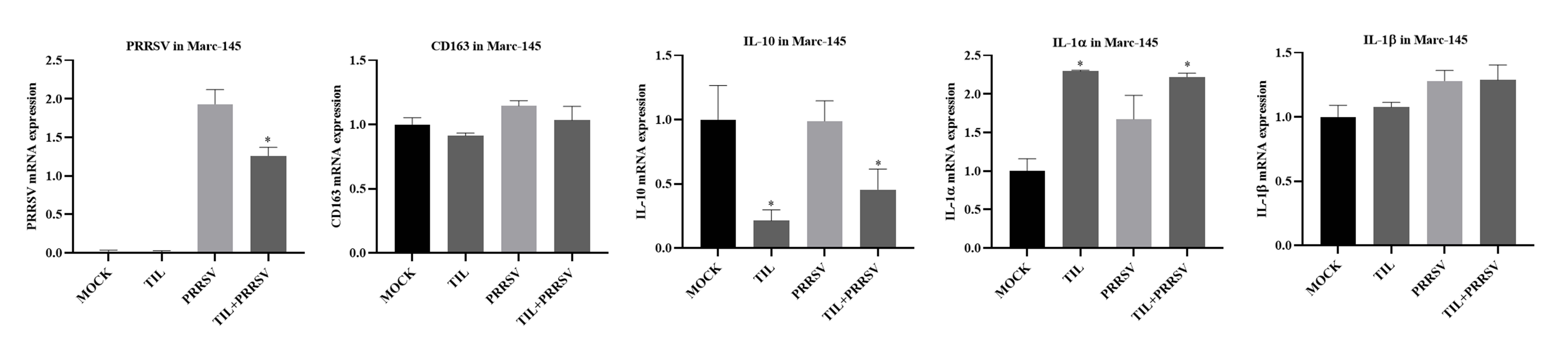

Supplement: Figure_S3_final.tiff [file KVIR_A_2561831_SM3156.tiff]

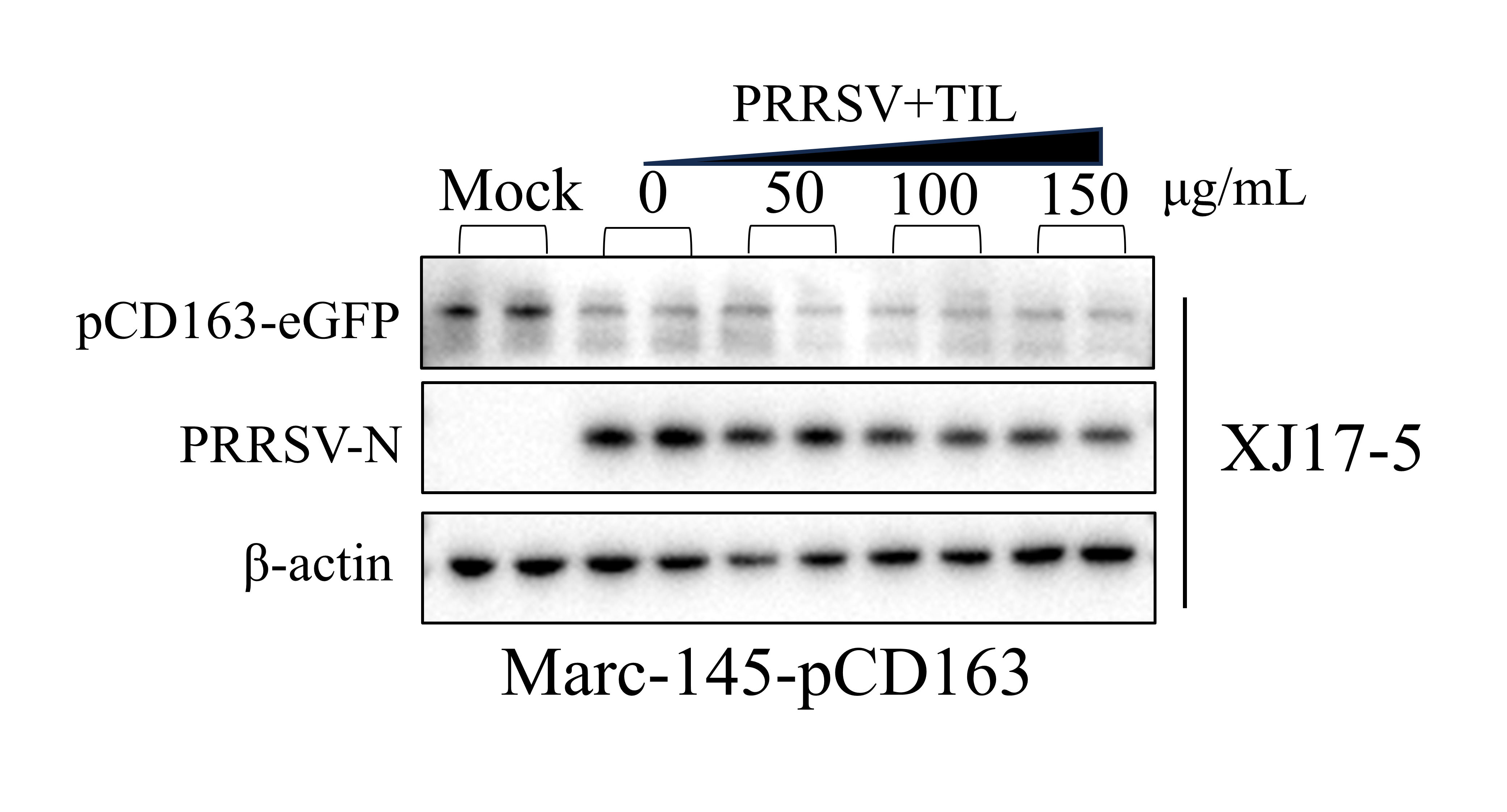

Supplement: Figure_S4_final.tiff [file KVIR_A_2561831_SM3154.tiff]

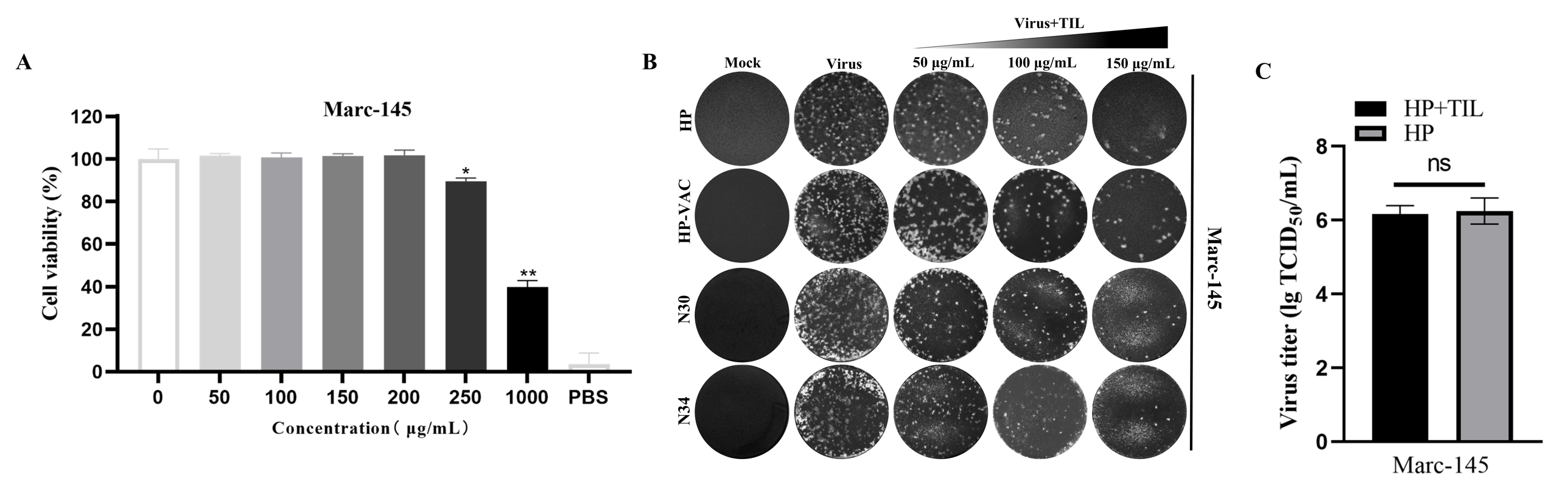

Supplement: Figure_S1_FINAL.tiff [file KVIR_A_2561831_SM3152.tiff]
