# Supplementary material for: Tilmicosin inhibits the infections of currently prevalent porcine reproductive and respiratory syndrome viruses via the downregulation of CD163 expression
Source: Virulence. 2025 Sep 15;16(1):2561831. doi: 10.1080/21505594.2025.2561831 (PMC12452467; doi:10.1080/21505594.2025.2561831)
Supplement: Supporting Table S1 primers.docx [file KVIR_A_2561831_SM3155.docx]

Table S1. Primers used in this study.

| No. | Target | Primer/Probe | Sequence (5’-3’) | Reference |
| --- | --- | --- | --- | --- |
| 1 | pCD163^*^ | pCD163-F | ATTCATCATCCTCGGACCCAT | This study |
| 2 |  | pCD163-R | CCCAGCACAACGACCACCT |  |
| 3 | pIL-10 | pIL-10-F | AGGATATCAAGGAGCACGTGAACT | This study |
| 4 |  | pIL-10-R | ACTCACCCATGGCTTTGTAGACA |  |
| 5 | pIL-1α | pIL-1α-F | AGATCAGCAACATACAGCTTCCAGA | This study |
| 6 |  | pIL-1α-R | TGACGGGTCTCGAATGATGCT |  |
| 7 | pIL-1β | pIL-1β-F | TGTCTGTGATGCCAACGTGC | This study |
| 8 |  | pIL-1β-R | TGTGAGGAGGTGGAGAGCCTT |  |
| 9 | pTLR8 | pTLR8-F | GAAACCGCCTGGACCTTTTG | Adapted from ([Ao et al., 2019](#_ENREF_1)) |
| 10 |  | pTLR8-R | GGGGCAAGTTAAGGAAGGCT |  |
| 11 | pCXCL10 | pCXCL10-F | CCCACATGTTGAGATCATTGC | This study |
| 12 |  | pCXCL10-R | CATCCTTATCAGTAGTGCCG |  |
| 13 | pMX1 | pMX1-F | AAGATAGTGGACGTGGCGAGAA | Adapted from ([Li et al., 2021](#_ENREF_2)) |
| 14 |  | pMX1-R | TCAAAGAAGGCCTGCTCCTTCT |  |
| 15 | pGAPDH | pGAPDH-F | CCTGCACCACCAACTGCT | Adapted from ([Ao et al., 2019](#_ENREF_1)) |
| 16 |  | pGAPDH-R | CACAGTCTTCTGGGTGGCA |  |
| 17 | mCD163^#^ | mCD163-F | TCTGTAATTTGCTCAGGAAACCAGT | This study |
| 18 |  | mCD163-R | AGCACAGCGACCTCCTCCAT |  |
| 19 | mIL-10 | mIL-10-F | AGCTGAGAACCACGACCCAGA | This study |
| 20 |  | mIL-10-R | TCACAGGGAAGAAATCGATGACA |  |
| 21 | mIL-1α | mIL-1α-F | GTTCTGAAGAAGAGACGGTTGAGT | This study |
| 22 |  | mIL-1α-R | TGGTATGTCATATTGCTTAGGAAGCT |  |
| 23 | mIL-1β | mIL-1β-F | AGTGTCTGAAGTAGCCATGGCA | This study |
| 24 |  | mIL-1β-R | TTAGCTTCTCCATGGCTACAACA |  |
| 25 | mGAPDH | mGAPDH-F | ATCACCATCTTCCAGGAGCGA | This study |
| 26 |  | mGAPDH-R | TGGTGGTGAAGACGCCAGTG |  |

* pCD163 indicates porcine CD163 same as the pIL-10, pIL-1α, pIL-1β, pTLR8, pCXCL10, pMX1 and pGAPDH.

# mCD163 indicates monkey CD163 same as the mIL-10, mIL-1α, mIL-1β and mGAPDH.
