# Supplementary material for: Tilmicosin inhibits the infections of currently prevalent porcine reproductive and respiratory syndrome viruses via the downregulation of CD163 expression
Source: Virulence. 2025 Sep 15;16(1):2561831. doi: 10.1080/21505594.2025.2561831 (PMC12452467; doi:10.1080/21505594.2025.2561831)
Supplement: Supporting Table S2 RNASeq quality.docx [file KVIR_A_2561831_SM3153.docx]

Table S2. RNA sequencing and data mapping of each sample.

| Group | Sample | Reads No. | Bases (bp) | Q20 (%) | Q30 (%) | Clean Reads No. | Clean Data (bp) | Clean Reads % | Total_Mapped | Uniquely_Mapped |
| --- | --- | --- | --- | --- | --- | --- | --- | --- | --- | --- |
| NEG | NEG_24_1 | 76297188 | 11444578200 | 97.7 | 93.79 | 70161760 | 10524264000 | 91.95 | 66479962 (94.75%) | 64346486 (96.79%) |
|  | NEG_24_2 | 45151044 | 6772656600 | 97.26 | 92.72 | 42294644 | 6344196600 | 93.67 | 40046594 (94.68%) | 38852288 (97.02%) |
| POS | N34_24P_1 | 62080200 | 9312030000 | 97.64 | 93.67 | 57091194 | 8563679100 | 91.96 | 53043917 (92.91%) | 51517917 (97.12%) |
|  | N34_24P_2 | 45231632 | 6784744800 | 97.37 | 93.07 | 41983592 | 6297538800 | 92.81 | 39010801 (92.92%) | 37880771 (97.10%) |
| TIL | N34_24T_1 | 63145832 | 9471874800 | 97.5 | 93.39 | 57767492 | 8665123800 | 91.48 | 54476262 (94.30%) | 52913154 (97.13%) |
|  | N34_24T_2 | 43418720 | 6512808000 | 97.54 | 93.35 | 40291252 | 6043687800 | 92.79 | 38137927 (94.66%) | 37017351 (97.06%) |
