# Supplementary material for: Tilmicosin inhibits the infections of currently prevalent porcine reproductive and respiratory syndrome viruses via the downregulation of CD163 expression
Source: Virulence. 2025 Sep 15;16(1):2561831. doi: 10.1080/21505594.2025.2561831 (PMC12452467; doi:10.1080/21505594.2025.2561831)
Supplement: Supplementary_figure_and_table_legends.docx [file KVIR_A_2561831_SM3151.docx]

**Figure legends:**

**Supplementary Figure S1. Tilmicosin inhibits PRRSV replication in Marc-145 cells.** (A) The cytotoxic effect of tilmicosin on Marc-145 cells was evaluated at 24 hpi using MTT assay. (B) Marc-145 cells were infected by 0.1 MOI of XJ17-5 (HP), JXA1-R (HP-VAC), rSD-VVL (N30) and rBJ-VVL (N34), and then treated with 50, 100 and 150 μg/mL tilmicosin, respectively. Viral plaques were visualized at 96 hpi. (C) Two groups of 100 μL HP-PRRSV-2 (XJ17-5) virus stocks (10^6^ TCID_50_) were incubated with 150 μg/mL TIL or with an equal volume of PBS, respectively. Both groups were gently agitated on a shaker at room temperature for 12 h. Subsequently, viral titers of these virus stocks were determined by TCID₅₀ assay. Statistical significances are denoted by *, *p* < 0.05; **, *p* < 0.01 (n=3). Data shown in bar graphs are means and standard deviations (SD).

**Supplementary Figure S2. Tilmicosin downregulates genes associated with antiviral innate immune responses.** The levels of TLR8, CXCL10 and MX1 were detected by transcriptomic sequencing (Left) and qPCR (right). Statistical significances are denoted by *, *p* < 0.05; **, *p* < 0.01 (n=3). Data shown in bar graphs are means and SD.

**Supplementary Figure S3. Tilmicosin modulates the expression of pro- and anti-inflammatory cytokines in Marc-145 cells.** HP-PRRSV2 XJ17-5 strain (0.1 MOI) was used to infect Marc-145 cells and 150 μg/mL tilmicosin was used for treatment. PRRSV ORF7 gene expression was detected by PRRSV universal real-time RT-PCR assay at 24 hpi. The CD163, IL-10, IL-1α, and IL-1β at 24 hpi were detected by qPCR using primers shown in Table S1. Statistical significances are denoted by *, *p* < 0.05; **, *p* < 0.01 (n=3). Data shown in bar graphs are means and SD.

**Supplementary Figure S4. Effect of TIL on PRRSV-N protein expression in Marc-145-pCD163 cells.** Marc-145 cells stably overexpressing pCD163 were treated with different concentrations of TIL for 12 hours. Cells were then infected with HP-PRRSV-2 (XJ17-5) isolate. At 24 hours post-infection (hpi), changes in the protein levels of PRRSV-N and pCD163-eGFP were analyzed by Western blotting (WB).

**Table legends:**

Table S1. Primers used in this study.

Table S2. RNA sequencing and data mapping of each sample.

Table S3. Top 20 differential expression genes in the TIL vs POS comparison
